# A review of outcome following valve surgery for rheumatic heart disease in Australia

**DOI:** 10.1186/s12872-015-0094-1

**Published:** 2015-09-23

**Authors:** E. Anne Russell, Lavinia Tran, Robert A. Baker, Jayme S. Bennetts, Alex Brown, Christopher M. Reid, Robert Tam, Warren F. Walsh, Graeme P. Maguire

**Affiliations:** 1 Baker IDI, Melbourne, VIC 3004 Australia; 2 School of Epidemiology and Preventive Medicine, Monash University, Melbourne, VIC Australia; 3 Department of Cardiac and Thoracic Surgery, Flinders Medical Centre, Adelaide, South Australia; 4 Department of Surgery, School of Medicine, Flinders University, Adelaide, South Australia; 5 Wardliparingga Aboriginal Research Unit, South Australia Health and Medical Research Institute, Adelaide, South Australia; 6 School of Population Health, University of South Australia, Adelaide, South Australia; 7 School of Public Health, Curtin University, Perth, Western Australia; 8 Director of Surgery, Department of Cardiothoracic Surgery, Townsville Hospital, Queensland, Australia; 9 Prince of Wales Hospital, Randwick, NSW Australia; 10 School of Medicine, James Cook University, Cairns, QLD Australia

**Keywords:** Indigenous health, Rheumatic heart disease, Rheumatic valve surgery, Outcome indicators

## Abstract

**Background:**

Globally, rheumatic heart disease (RHD) remains an important cause of heart disease. In Australia it particularly affects younger Indigenous and older non-Indigenous Australians. Despite its impact there is limited understanding of the factors influencing outcome following surgery for RHD.

**Methods:**

The Australian and New Zealand Society of Cardiac and Thoracic Surgeons Cardiac Surgery Database was analysed to assess outcomes following surgical procedures for RHD and non-RHD valvular disease. The association with demographics, co-morbidities, pre-operative status, valve(s) affected and operative procedure was evaluated.

**Results:**

Outcome of 1384 RHD and 15843 non-RHD valve procedures was analysed. RHD patients had longer ventilation, experienced fewer strokes and had more readmissions to hospital and anticoagulant complications. Mortality following RHD surgery at 30 days was 3.1 % (95 % CI 2.2 – 4.3), 5 years 15.3 % (11.7 – 19.5) and 10 years 25.0 % (10.7 – 44.9). Mortality following non-RHD surgery at 30 days was 4.3 % (95 % CI 3.9 - 4.6), 5 years 17.6 % (16.4 - 18.9) and 10 years 39.4 % (33.0 - 46.1). Factors independently associated with poorer longer term survival following RHD surgery included older age (OR1.03/additional year, 95 % CI 1.01 – 1.05), concomitant diabetes (OR 1.7, 95 % CI 1.1 – 2.5) and chronic kidney disease (1.9, 1.2 – 2.9), longer invasive ventilation time (OR 1.7 if greater than median value, 1.1– 2.9) and prolonged stay in hospital (1.02/additional day, 1.01 – 1.03). Survival in Indigenous Australians was comparable to that seen in non-Indigenous Australians.

**Conclusion:**

In a large prospective cohort study we have demonstrated survival following RHD valve surgery in Australia is comparable to earlier studies. Patients with diabetes and chronic kidney disease, were at particular risk of poorer long-term survival. Unlike earlier studies we did not find pre-existing atrial fibrillation, being an Indigenous Australian or the nature of the underlying valve lesion were independent predictors of survival.

## Background

Rheumatic heart disease (RHD) is a condition of global health importance. It is estimated 15.6 to 19.6 million people are living with RHD, with almost 80 % of these residing in low and middle-income countries [1, 2], with an estimated population prevalence in those countries of 2.5 to 3.2 cases per 1000 [1]. Approximately 1 to 5 % of people with RHD die each year accounting for 233 000 to 294 000 RHD-related deaths per year, 95 % of these occurring in low- and middle income countries [1] with limited facilities to treat advanced disease requiring valve surgery.

Whilst RHD is now rare in high income countries [3], except for migrant and older residents, it remains an important and ongoing cause of preventable heart disease in Indigenous populations [4]. A recent echocardiographic screening study of Indigenous Australian (Aboriginal and Torres Strait Islander) children aged 5–14 years, found a prevalence of RHD [5] of 8.6 per 1000 (95 % CI 6.0 – 12.0) with none detected in a comparably aged non-Indigenous cohort [6].

Surgical intervention remains an important treatment modality for those with more severe forms of RHD, yet disparities exist in access to and outcomes following RHD surgery [7]. Factors which have been identified as being associated with outcomes following valve surgery in patients with RHD-related valve disease include age [8–11], pre-operative clinical status [8–10, 12–15], pre-existing atrial fibrillation (AF) [13, 16, 17], left ventricular function [12–14, 18, 19] and the nature of the underlying valve lesion [10, 12, 13, 20].

Increasing age has been associated with lower overall event-free survival [8–11, 21] and operative mortality [16]. Younger patients are, presumably due to longer overall survival, nonetheless subject to a higher risk of eventual deterioration of bioprosthetic valves, with an attendant need for reoperation [19, 22–25]. Other factors which have been reported as being associated with outcome following RHD-related valve surgery include poorer pre-operative clinical status, as assessed by New York Heart Association (NYHA) functional class [26–28] and impaired pre-operative left ventricular function (left ventricular ejection fraction (LVEF) <45 %) [9, 10, 19]. Preoperative AF has also been found to predict later mortality [16, 26, 29]. Finally the valve involved and the nature of the valve lesion (regurgitation versus stenosis) has been shown to influence outcome with the best long-term outcome seen in those with isolated mitral regurgitation [29].

It has been suggested Indigenous Australians (Aboriginal Australians and Torres Strait Islander peoples) may have poorer survival following RHD valve surgery compared with non-Indigenous Australian patients [16, 25, 26]. Nonetheless previous studies have tended to suffer from a lack of power, have usually been restricted to single site and often failed to control for other factors which may influence survival. Despite tending to be younger at time of surgery, Indigenous Australians have previously been found to have poorer survival within the first 30 days following valve surgery [16, 26] and at five years [16, 26, 30]. Where disparities have been noted they have been attributed to a range of factors including comorbidities [16, 25, 26], barriers to primary and specialist health care and access, compliance and monitoring of anticoagulation during long-term follow-up [23].

Whilst existing national Australian guidelines [25] for RHD management acknowledge that outcomes may be affected by treatment choice, prosthetic valve type and timing of referral for intervention, there remains limited information regarding how these factors interact and how they might be anticipated to influence outcomes and treatment recommendations.

We therefore aimed to identify factors associated with RHD surgery outcome by analysing data from a large multi-site cardiac surgery enhanced surveillance register, The Australia and New Zealand Society of Cardiac and Thoracic Surgeons (ANZSCTS) National Cardiac Surgery Database.

## Methods

### The Database

The Australia and New Zealand Society of Cardiac and Thoracic Surgeons (ANZSCTS) National Cardiac Surgery Database is an Australia-wide voluntary database for the prospective collection and analysis of the results of cardiac surgery. It collects data from 25 Australian hospitals on patients who have undergone cardiac surgery, the types of surgery performed and early (30 day) complications [31–33] and links this with long-term survival data.

### Analysis

Demographic data including age, gender, location and Indigenous status were assessed. The remoteness of the usual place of residence was classified based on the Australian Statistical Geography Standard [34] as Remote (Remoteness Area (RA) categories 3 or 4) or non-Remote. Co-morbidities assessed included chronic kidney disease (defined as pre-operative estimated glomerular filtration rate (eGFR) less than 60 mL/min/1.73 m^2^ based on the Modification of Diet in Renal Disease (MDRD) equation [35] and stratified to stages 3 (30 – 59 mL/min/1.73 m^2^), 4 (15 – 29 mL/min/1.73 m^2^), and 5 (<15 mL/min/1.73 m^2^) [37], previous and current smoking status, concomitant coronary artery bypass grafting (CABG) and a pre-existing clinician diagnosis of diabetes mellitus and hypertension. The pre-operative status relating to underlying heart disease included New York Heart Association (NYHA) classes I to IV [37], pre-operative atrial fibrillation (AF), echocardiographic assessment of LVEF stratified to more than 45 %, 30 to 45 % or less than 30 % and previous percutaneous balloon valvuloplasty (PBV) or valve surgery.

Valvular lesions were analysed according to the type and number of valve(s) affected. Valve-related surgical procedure data included valve repair or replacement and in the case of replacement, whether this was a mechanical or bioprosthetic valve.

Outcomes associated with the immediate post- operative course included length of time of invasive ventilatory support and length of intensive care stay (expressed as dichotomous variables based on median values), hospital length of stay in days and the need for re-operation during the initial admission. Early outcomes within the 30 days following surgery included mortality, stratified as cardiac and non-cardiac, readmission and other complications (valve dysfunction, acute kidney injury, new atrial fibrillation, stroke/TIA, deep sternal wound infection, septicaemia, anticoagulation (bleeding, and/or embolic) complications and heart failure). Finally longer-term survival beyond 30 days was determined from the National Death Index (NDI), a database, housed at the Australian Institute of Health and Welfare, which contains records of all deaths occurring in Australia since 1980 [38].

### Statistical analysis

Data were analysed using IBM SPSS Statistics 20 (IBM, New York, USA) and STATA Release 13 (StataCorp LP, Texas, USA). Descriptive data were summarised using standard univariate techniques and reported as percentages with 95 % confidence intervals (95 % CI), means with standard deviation (SD) or medians with interquartile range (IQR) depending on the data format and distribution. Comparisons between groups were undertaken using χ^2^ for categorical data and Student’s *t*-Test or Mann–Whitney *U* test for continuous Normally distributed or non-Normally distributed data respectively. A p valve less than 0.05 was taken to indicate statistical significance and all tests were two-sided.

Survival analysis for mortality was presented with Kaplan- Meier curves and analysed using the log rank test to compare survival in RHD and non-RHD surgery and Indigenous and non-Indigenous Australian RHD patients.

Multivariable linear, logistic and Cox proportional hazard models were developed to identify independent factors associated with outcome measures. These used a backwards stepwise approach including in the first model all factors associated with a particular outcome variable using bivariate analysis with a p value <0.1. Factors with a *p* value > =0.05 were progressively removed from the models starting with those variables with a regression co-efficient closest to 0 or an odds (OR) or hazard (HR) ratio closest to 1. Final models were limited to predictive factors with significant coefficients (*p* < 0.05).

Approval for this project was granted by the Monash University Human Research Ethics Committee (CF13/2737 – 2013001472).

## Results

Data in relation to 62 707 cardiac surgical procedures were collated by the ANZCTS database between 1 June 2001 and 31 December 2012. Details regarding the breakdown of patients included in this database have been outlined elsewhere [33]. A subset of 17 227 surgical valve procedures (with or without coronary artery bypass grafting (CABG)) was included for analysis. Demographic and comorbidity data relating to these patients are outlined in Table 1. RHD was a significantly more common indication for valve surgery in Indigenous (52.4 %, 95 % CI 46.9 – 57.9) as compared with non-Indigenous Australians (7.2 %, 95 % CI 6.8 – 7.6 %) (*p* <0.001).

**Table 1:** Descriptive characteristics of valve surgery patients stratified by whether indication for surgery was RHD or non-RHD related [33]

	All	RHD-related	Non-RHD	*P* value
	*N* = 17227	*N* = 1384	*N* = 15843	
Age (years) (median (IQR^a^)	71.3(61.2 – 78.3)	59.7(50.9 – 71.4)	71.9(62.3 – 78.6)	<0.001
Sex (% female) (95 % CI^#^)	37.3(36.6 – 38.1)	64.5(61.9 – 67.0)	35.0(34.2 – 35.7)	<0.001
Indigenous status (% Aboriginal and Torres Strait Islander people) (95 % CI)	1.9(1.7 – 2.1)	12.6(10.9 – 14.4)	1.0(0.8 – 1.2)	<0.001
Concomitant CABG (%, 95 % CI)	39.1(38.4 – 39.8)	21.2(19.1 – 23.5)	40.7(39.9 – 41.4)	<0.001
Pre-operative comorbidities				
Diabetes (%, 95 % CI)	23.2(22.5 – 23.8)	20.3(18.2 – 22.5)	23.4(22.8 – 24.1)	0.009
Chronic kidney disease (% eGFR < 60 mL/min/1.73 m^2^) (95 % CI))	36.7(36.0 – 37.5)	31.2(28.8 – 33.7)	37.2(36.5 – 38.0)	0.814
Hypertension (%, 95 % CI)	67.0(66.3 – 67.7)	53.0(50.3 – 55.7)	68.2(67.5 – 68.9)	<0.001
Previous smoking (%, 95 % CI)	53.1(52.3 – 53.8)	52.7(50.0 – 55.3)	53.1(52.3 – 53.9)	0.955
Current smoking (%, 95 % CI)	16.0(15.2 – 16.7)	25.1(22.0 – 28.4)	15.2(14.5 – 16.0)	<0.001
Pre-operative status				
NYHA classes III & IV (%, 95 % CI)	43.7(42.9 – 44.4)	53.7(51.0 – 56.4)	42.8(42.0 – 43.6)	0.351
Atrial fibrillation (%, 95 % CI)	19.3(18.7 – 19.9)	40.5(37.9 – 43.2)	17.4(16.8 – 18.0)	<0.001
LVEF >45 % (%, 95 % CI)	81.2(80.6 – 81.8)	84.6(82.6 – 86.5)	80.9(80.3 – 81.5)	0.001
LVEF 30 – 45 % (%, 95 % CI)	12.1(11.6 – 12.6)	10.9(9.3 – 12.7)	12.2(11.7 – 12.7)	0.154
LVEF <30 % (%, 95 % CI)	4.3(4.0 – 4.6)	2.2(1.5 – 3.2)	4.5(4.2 – 4.8)	<0.001
Previous procedures				
Valve surgery (%, 95 % CI)	6.4(6.1 – 6.8)	13.5(11.8 – 15.4)	5.8(5.4 – 6.2)	<0.001
Percutaneous balloon valvuloplasty (PBV) (%, 95 % CI)	4.9(4.3 – 5.6)	20.7(16.7 – 25.2)	3.3(2.8 – 4.0)	<0.001

^a^IQR – interquartile range, ^#^95 % CI – 95 % confidence intervals

Over a maximum period of follow-up of 10.5 years there were 2089 deaths reported, 157 in RHD patients (11.3 %) and 1932 in non-RHD patients (12.2 %). Data regarding crude 30 day, 5 year and 10 year survival stratified by RHD or non-RHD valve surgery are presented in Table 2.

**Table 2:** Unadjusted mortality at 30 days, 5 years and 10 years stratified by RHD or non-RHD valve surgery

Mortality number (%, 95 % CI)	All valve surgery	RHD valve surgery	Non-RHD valve surgery	RR* (95 % CI) (*p* value)
30 day	576/13866(4.2, 3.8 – 4.5)	35/1137(3.1, 2.2 – 4.3)	541/12729(4.3, 3.9 – 4.6)	1.38 (0.99 – 1.93)(0.058)
5 year	665/3821(17.4, 16.2 – 18.6)	54/353(15.3, 11.7 – 19.5)	611/3468(17.6, 16.4 – 18.9)	1.15 (0.89 – 1.49)(0.273)
10 year	96/254(37.8, 31.8 – 44.1)	7/28(25.0, 10.7 – 44.9)	89/226(39.4, 33.0 – 46.1)	1.58 (0.81 – 3.05)(0.139)

*RR - Relative risk in RHD-related valve surgery patients compared with non-RHD surgery

### 30 day outcomes

Outcomes within 30 days following surgery are outlined in Table 3. RHD patients, compared with non-RHD patients, had a longer period of invasive ventilation and a higher rate of readmission to hospital but no difference in 30 day survival. RHD patients were less likely to have a stroke but were more likely to have an anticoagulant complication.

**Table 3:** Outcome of valve surgery within 30 days

	All	RHD-related	Non-RHD	*P* value
	*N* = 17227	*N* = 1384	*N* = 15843	
Initial admission				
Ventilation (hours) (median (IQR))	11.0(6.8 – 19.0)	12.0(7.0 – 19.0)	11.0(6.7 – 19.0)	0.009
Intensive care unit (ICU) stay (hours) (median (IQR))	43.3(23.0 – 72.3)	42.0(23.0 – 70.8)	43.5(23.0 – 72.5)	0.350
Post procedure length of stay (days) (median (IQR))	8.0(7.0 – 13.0)	8.0(7.0 – 13.0)	8.0(7.0 – 13.0)	0.648
Re-operation for valve dysfunction (%, 95 % CI)	0.2(0.1 – 0.3)	0.4(0.1 – 0.8)	0.2(0.1 – 0.3)	0.152
Re-operation not related to valve dysfunction (%, 95 % CI)	7.0(6.6 – 7.4)	7.3(6.0 – 8.8)	7.0(6.6 – 7.4)	0.652
Mortality				
All cause (%, 95 % CI)	4.2(3.8 – 4.5)	3.1(2.2 – 4.3)	4.3(3.9 – 4.6)	0.058
Cardiac cause (%, 95 % CI)	1.5(1.3 – 1.8)	1.5(0.9 – 2.4)	1.5(1.3 – 1.8)	0.122
Non-cardiac cause (%, 95 % CI)	2.7(2.4 – 2.9)	1.6(1.0 – 2.6)	2.8(2.5 – 3.0)	0.907
Readmission (%, 95 % CI)	11.2(10.7 – 11.7)	13.8(12.0 – 15.7)	11.0(10.5 – 11.5)	0.002
Other complications				
Readmission for valve dysfunction (%, 95 % CI)	0.2(0.1 – 0.4)	0	0.2(0.1 – 0.4)	0.205
Acute kidney injury (%, 95 % CI)	6.3(5.9 – 6.7)	6.3(5.1 – 7.7)	6.3(5.9 – 6.7)	0.971
New AF (% without prior AF, 95 % CI)	34.2(33.4 – 35.1)	33.3(30.1 – 36.6)	34.3(33.5 – 35.1)	0.564
Stroke/ TIA (%, 95 % CI)	2.4(2.2 – 2.6)	1.6(1.0 – 2.4)	2.5(2.2 – 2.7)	0.044
Deep sternal wound infection (%, 95 % CI)	0.9(0.8 – 1.1)	1.2(0.7 – 1.0)	0.9(0.8 – 1.1)	0.247
Anticoagulant complication (bleeding or embolization) (%, 95 % CI)	1.7(1.5 – 1.9)	2.8(2.0 – 3.7)	1.6(1.4 – 1.8)	0.002
Heart failure (%, 95 % CI)	1.9(1.5 – 2.2)	2.4(1.4 – 3.9)	1.8(1.5 – 2.1)	0.274
Septicaemia (positive blood culture with signs of infection)(%, 95 % CI)	1.6(1.4 – 1.8)	0.4(0.8 – 2.1)	0.6(1.4 – 1.8)	0.476

Factors independently associated with 30 day mortality following valve surgery using logistic regression modelling are listed in Table 4.

**Table 4:** Factors independently associated with 30 day mortality following valve surgery in logistic regression modelling and variance explained by the model

Odds Ratio (95 % CI)	All	RHD-related	Non-RHD
Age (/additional year)	1.01 (1.00 – 1.02)	-	-
Female sex	-	-	1.4 (1.1 – 1.8)
Pre-operative comorbidities			
Chronic kidney disease	2.4 (1.8 – 3.2)	4.3 (2.0 – 9.2)	2.6 (2.0 – 3.3)
Pre-operative status			
NYHA III & IV	1.7 (1.3 – 2.1)	-	1.7 (1.3 – 2.2)
LVEF 30 – 45 %	2.4 (1.8 – 3.2)	-	2.4 (1.8 – 3.3)
LVEF <30 %	3.5 (2.4 – 5.1)	-	3.6 (2.5 – 5.3)
Mitral valve regurgitation	1.2 (1.1 – 1.3)	-	1.2 (1.1 – 1.2)
Mitral valve stenosis	0.9 (0.8 – 0.9)	-	0.9 (0.8 – 0.9)
Previous procedures			
Valve surgery	2.4 (1.6 – 3.4)	2.5 (1.1 – 5.8)	2.4 (1.6 – 3.5)
Initial admission			
ICU stay (>43 h)	0.7 (0.6 – 0.9)	0.3 (0.1 – 0.7)	-
Post procedure LOS (/additional day)	0.97 (0.96 – 0.98)	-	0.96 (0.95 – 0.97)
Complications within 30 days			
Readmission	0.4 (0.3 – 0.7)	-	0.4 (0.2 – 0.6)
Re-operation for valve dysfunction	-	27.5 (2.2 – 338.9)	-
Re-operation not related to valve dysfunction	2.7 (2.0 – 3.8)	3.3 (1.3 – 8.4)	2.8 (2.2 – 3.9)
Acute kidney injury	7.3 (5.5 – 9.8)	7.3 (2.9 – 17.9)	6.9 (5.1 – 9.3)
Stroke/ TIA	4.6 (2.9 – 7.2)	-	4.6 (2.8 – 7.4)
Anticoagulant complication	2.9 (1.7 – 5.1)	-	2.8 (1.5 – 5.1)
Septicaemia	9.5 (6.1 – 15.27)	9.0 (2.5 – 32.3)	10.2 (6.3 – 16.4)
Explained variance			
(Nagelkerke R Square statistic [68])	24.6 %	24.7 %	25.1 %

### Long term survival

**Fig. 1:**
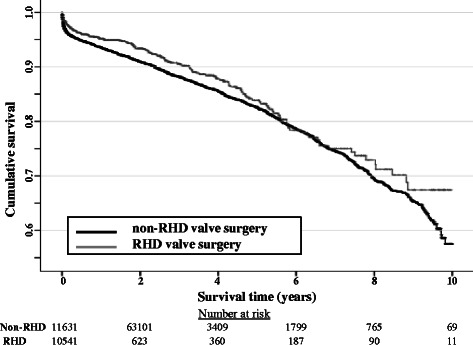
Cumulative survival following RHD and non-RHD-related valve surgery (log rank test, significant *p* = 0.047)

Kaplan-Meier curves comparing mortality in RHD and non-RHD-related valve surgery are shown in Fig. 1. Log rank testing of mortality in RHD and non-RHD patients demonstrated a small, but statistically, significant difference in survival out to 10 years with superior survival in RHD valve surgery patients.

Factors independently associated with longer term mortality following valve surgery using Cox proportional modelling are outlined in Table 5.

**Table 5:** Factors independently associated with long term mortality following valve surgery in Cox proportional hazard modelling and the significance of the relationship of the model

Hazard Ratio (95 % CI)	All	RHD-related	Non-RHD
Age (/additional year)	1.03 (1.02 – 1.04)	1.03 (1.01 – 1.05)	1.03 (1.02 – 1.04)
Pre-operative comorbidities			
Diabetes	1.4 (1.2 – 1.6)	1.7 (1.1 – 2.5)	1.4 (1.2 – 1.6)
Chronic kidney disease	1.5 (1.3 – 1.7)	1.9 (1.2 – 2.9)	1.4 (1.3 – 1.6)
Pre-operative status			
NYHA III & IV	1.3 (1.1 – 1.4)	-	1.3 (1.1 – 1.4)
Atrial fibrillation	1.4 (1.2 – 1.6)	-	1.5 (1.3 – 1.7)
LVEF >45 %	0.7 (0.6 – 0.8)	-	0.7 (0.6 – 0.8)
Operative procedure			
Mechanical valve	0.8 (0.7 – 0.9)	-	0.8 (0.7 – 0.9)
Valve repair only	0.8 (0.6 – 0.9)	-	0.8 (0.6 – 0.9)
Multiple valve surgery	-	-	1.4 (1.1 – 1.7)
Initial admission			
ICU stay (>43 h)	1.2 (1.1 – 1.4)	-	1.3 (1.1 – 1.4)
Ventilation time (>11 h)	-	1.7 (1.1 – 2.9)	-
Post procedure LOS (/additional day)	1.01 (1.00 – 1.01)	1.02 (1.01 – 1.03)	1.00 (1.00 – 1.01)
Complications within 30 days			
Readmission	1.4 (1.2 – 1.6)	-	1.4 (1.2 – 1.7)
Acute kidney injury	1.9 (1.6 – 2.3)	-	1.9 (1.6 – 2.3)
Stroke/ TIA	1.6 (1.2 – 2.1)	-	1.7 (1.3 – 2.2)
Septicaemia	2.1 (1.6 – 2.8)	-	2.2 (1.6 – 2.9)
Significance of model (based on −2 Log Likelihood)	<0.001	<0.001	<0.001

Of note was, once these factors were controlled for, the superior longer term survival associated with RHD was no longer present. In addition, being Indigenous Australian, the nature of the valve lesion and the presence of poorer preoperative LVEF were not independently associated with longer-term survival in RHD patients after controlling for the factors highlighted in Table 5.

### Outcome in Indigenous Australians

Indigenous RHD patients, compared with non-Indigenous RHD patients had a shorter post procedural length of hospital stay (7 days (95 % CI 6.0 – 10.0) compared to 8 days (95 % CI 7.0 – 12.0)) and were less likely to develop acute kidney injury (2.9 % (95 % CI 1.0 – 6.7) compared to 6.8 % (95 % CI 5.4 – 8.4)) or AF post-operatively (13.8 % (95 % CI 8.1 – 21.4) compared to 36.5 % (95 % CI 32.9 – 40.2)).

Thirty day mortality following RHD valve surgery in Indigenous Australians was comparable to that seen in non-Indigenous Australians (2.9 % compared with 3.1 %, *p* = 0.895). On logistic regression modelling restricted to Indigenous Australians only two factors were independently associated with 30 day mortality in those having RHD valve surgery: chronic kidney disease (OR 14.1, 95 % CI 1.0 – 200.0) and readmission (OR 20.8, 95 % CI 1.5 – 333.3).

Longer term mortality following RHD surgery was also comparable in Indigenous and non-Indigenous patients (10.3 % compared with 11.5 %, *p* = 0.657). Three factors were independently associated with longer term mortality in Indigenous Australians using Cox proportional modelling: LVEF <30 % (HR 31.3, 95 % CI 7.0 – 142.9), a longer period of ventilation (1.04/additional hour, 95 % CI 1.01 – 1.07), and a shorter initial stay in hospital (0.5/additional day, 95 % CI 0.3 – 0.8).

Remote location was not a significant predictor of either short or long term outcome either alone or in association with procedure type (log rank test, *p* = 0.594) in Indigenous Australians, who were more likely to reside in such locations.

### Outcome and procedure type

**Fig. 2:**
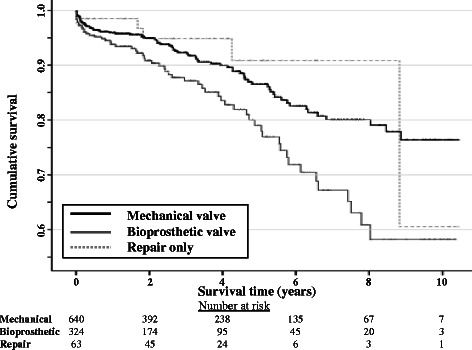
Cumulative survival following RHD-related valve surgery stratified by procedure type (log rank test, *p* = 0.001)

The relationship between the type of surgical procedure for RHD-related disease and survival was analysed over a maximum period of follow-up of 10.5 years. There were 33 (11.8 %, 95 % CI 8.3 – 16.2, *p* = 0.775) deaths reported following RHD-related valve repair (five for isolated repair without associated other valve surgery), 65 (14.1 %, 95 % CI 11.0 – 17.6, *p* = 0.024) following RHD-related bioprosthetic valve replacement (58 for isolated bioprosthetic valve replacement) and 84 (10.1 %, 95 % CI 8.2 – 12.4, *p* = 0.082) (76 for isolated mechanical valve replacement) following RHD-related mechanical valve replacement. A Kaplan-Meier curve comparing mortality in RHD-related valve repair, bioprosthetic valve and mechanical valve surgery is shown in Fig. 2 and demonstrated a significant difference in survival between operative groups. This difference specifically related to poorer survival following bioprosthetic replacement (HR 1.5 (95 % CI 1.1 – 2.0). Multivariate survival analysis for these RHD patients (see Table 5) demonstrated this difference in survival persisted after controlling for co-existent diabetes and chronic kidney disease, performance status, ventilation time, hospital length of stay and early septicaemia.

## Discussion

This is the largest published study of short and longer-term outcome following RHD valve surgery in Australia. Whilst rheumatic valve surgery was relatively uncommon, representing only 8 % of all valve surgery procedures performed during the study period, it represented a significant proportion (>50 %) of valve procedures in Indigenous Australians. Such findings highlight the higher burden of RHD in Indigenous Australians. Nonetheless the finding that 7.2 % of valve procedures in non-Indigenous Australians were for RHD-related valve disease also demonstrates the remaining importance of residual, and particularly advanced, RHD in non-Indigenous Australians who accounted for the greatest overall number of patients. Much of this RHD in non-Indigenous Australians was presumably associated with residents who had immigrated to Australia from countries where RHD remained endemic or who had acquired RHD decades before, at a time when acute rheumatic fever (ARF) remained an issue for all Australians, rather than predominantly Indigenous Australians as is the case now [6].

Whilst RHD is a relatively common indication for valve surgery, it is not a major contributor to overall mortality in Australia. Nationally, between 2007 and 2009, there were only 897 deaths registered with RHD as the primary cause of death. This accounted for 0.6 % of cardiovascular and 0.2 % of all deaths [40]. National data nonetheless do not highlight the particular impact RHD has on Indigenous Australians. Whilst between 2004 and 2007 there were only 63 deaths from RHD among Indigenous Australians (5.8 per 100,000 population) this rate was 5.2 times greater than that for non-Indigenous Australians (1.1 per 100,000 population) [39].

Our study highlights that survival following valve surgery in the short (30 days) and longer term is equivalent in RHD and non-RHD patients. This concurs with Ribeiro et al’s recent review of 352 Brazilian patients who underwent mitral valve replacement. In their study RHD was an indication in 43.5 % of patients and, in similar multivariate analysis, they demonstrated no significant difference in long-term survival for RHD-related surgery [40]. Dillon et al’s Malaysian study of mitral valve repair in RHD and non-RHD patients [41] also demonstrated no difference in short and long term survival between these groups. Our Australian valve surgery patients also had short and long-term survival that was equivalent to earlier cohorts studies of aortic and mitral valve replacement and repair. Chiang et al’s US study of survival following aortic valve replacement [42] found an equivalent 30 day mortality of 3 % and Dillon et al’s Malaysian study of mitral valve repair [41] a comparable mortality of 4.3 % in RHD patients and 2.0 % in non-RHD patients. The long-term (10 year) survival found in our study (88.7 %) was at the upper limit reported by other studies including Chiang (76 %) [42], Dillon (83-89 %) [41] and Ribeiro (71-74 %) [40]. Neither short nor long term survival was significantly related to Indigenous status as has been suggested in a previous study [26].

A range of other factors had also been identified as being associated with outcome following surgery for advanced RHD [22–24, 43]. These encompassed factors associated with the underlying severity of valve disease [10, 12, 13, 20, 29, 44–48], the procedure undertaken [8–10, 18, 25, 49–57], social and environmental factors that may have increased the risk of ARF/RHD and the risk of complications (e.g. social and environmental disadvantage including access to initial surgical and ongoing primary and specialist health care review) and patient factors that were independent of RHD (e.g. age and comorbidities) [8–11, 16, 21, 25, 46, 47, 49, 58–60]. In contrast our study found that many of these factors were not significant predictors of subsequent short and long term survival in this large cohort using multivariable analysis.

In our study, RHD valve surgery patients, compared to those having valve surgery for non-RHD indications, were more than twice as likely to have pre-operative AF. This has previously been found to significantly increase the risk of late death [13, 16, 29, 46] especially from cardioembolic complications [17]. This greater level of AF in RHD patients has been reported in previous studies including in Dillon et al’s review of RHD and non-RHD related valve repair in Malaysia which found 36 % of RHD patients undergoing mitral valve repair had pre-operative AF compared with 25 % of non-RHD patients [41]. Whilst we demonstrated similar levels of preoperative and post-operative AF, unlike previous studies, neither prior nor new post post-operative AF was an independent predictor of survival. Although this difference may relate to superior long-term anticoagulation in our setting it was not possible to confirm this based on the lack of long-term post-operative anticoagulation results in our cohort.

The greater risk of pre-operative AF in our patients with advanced RHD would nonetheless suggest there may be differences in the atria between RHD and non-RHD patients at the time of surgery. Whether this relates to more advanced valvular dysfunction with attendant increased left atrial volume [61] or other influences on atrial conduction [62] remains to be seen. Irrespective of its underlying aetiology and influence on overall survival, this increased burden of pre-operative AF, will necessarily translate to an attendant greater need, risk and inconvenience of anticoagulation in some patients and has been shown to be associated with surgical choice [33].

Under and over anticoagulation following valve surgery is common [22, 43, 49, 63] and has been associated with thromboembolism, bleeding [1, 30] and poorer survival [12]. In general, anticoagulation can be suboptimal in all patient groups, and RHD valve surgery patients in this study were more likely, compared with non- RHD patients, to develop an anticoagulant complication. This appeared to be particularly related to bleeding rather than the cardioembolic complications of stroke or TIA. This lesser risk of stroke and greater risk of other anticoagulant complications would suggest monitoring and titration of anticoagulation, rather than medication adherence, is a more important contributor to early post-operative complications in our RHD patients. More detailed understanding of the adequacy of early post-operative anticoagulation monitoring and treatment titration in RHD valve surgery patients will be required to understand and potentially minimize this increased risk.

Increasing age has been shown, in previous studies, to be associated with poorer survival [9–11, 21, 47, 58] and an increased need for reoperation [59]. The greater burden of RHD in younger Indigenous patients has been highlighted and whilst a younger age at the time of RHD surgery did have an independent effect on survival following surgery, Indigenous status did not. Such younger patients are likely to be eventually at risk of structural valve deterioration with an attendant greater need for reoperation [29].

Whilst we could not report on the eventual need for reoperation in our cohort it is reassuring that in other studies this risk is relatively small, being required at 10 years in 1.6 % of RHD patients having mitral valve repair [41], 7.3 % of RHD and non-RHD patients with mechanical mitral valve replacement [40] and 13.6 % of RHD and non-RHD patients with bioprosthetic mitral valve replacement [40]. In a setting where late reoperation might be expected to be required in up to 15 % of often younger Indigenous RHD patients it was noted that such reoperation was associated with increased perioperative mortality but equivalent longer term survival.

In earlier studies objective (LVEF) and functional (NYHA) measures of cardiac function have both been associated with outcome following valve surgery [13, 14, 19]. In this study the adverse impact of poorer LVEF and NYHA on short-term survival was demonstrated when the outcome of all valve surgery was analysed but not when this was restricted to RHD patients alone. The failure to demonstrate such an influence in RHD-related surgery may have been related to our use of multivariate survival analysis. Poorer LVEF was nonetheless found to adversely impact longer term survival when analysis was restricted to Indigenous Australian patients, perhaps highlighting how communication and accurate assessment of performance status may be difficult in a setting of cultural and linguistic diversity.

The importance of NYHA functional class as an independent predictor of survival in the short (perioperative) [12–14, 27, 28] and longer term [8–10, 14, 47] has been demonstrated by numerous studies. Our finding that poorer preoperative clinical status, based on NYHA class, was not independently associated with longer term mortality may suggest other cardiac and non-cardiac factors that influence NYHA-measured function, such as unreported or identified pulmonary hypertension or undiagnosed coronary heart disease, may have had an independent effect on survival. Functional assessment prior to surgery would therefore appear to have an important ongoing role in predicting outcome of surgery in addition to other investigations.

Following discharge, RHD valve surgery patients were more likely to be readmitted to hospital compared with non-RHD valve surgery patients. Although not explicitly recorded, persistent or recurrent rheumatic carditis may have been important in this setting as both are significant factors associated with valve replacement [19] and repair failure [12]. This in part provides the rationale for the recommendation for long-term secondary antibiotic prophylaxis following surgery even if the risk of recurrent ARF is deemed to be low [22, 25, 64].

Chronic diseases were frequent co-morbidities in patients having RHD and non-RHD surgery. Nonetheless it was only chronic kidney disease that was associated with 30 day mortality in both RHD and non-RHD patients and more specifically, Indigenous Australians. Chronic kidney disease and diabetes were both associated with poor longer term survival in RHD and non-RHD patients. The adverse effect of kidney disease on post-operative survival [46, 58] is well described. In Australia between 2007 and 2009 19 % of people dying from RHD had kidney disease as a contributing factor [39]. The association between co-existent diabetes and kidney disease, conditions commonly seen in Aboriginal and Torres Strait Islander patients and older Australians, and outcome following valve surgery highlights how changing disease profiles in an ageing Australian population may influence trends in valve surgery outcomes.

### Limitations of the study

The multicenter nature of this study poses potential limitations. We have shown a number of differences between RHD and non-RHD valve surgery patients and factors associated with short and longer-term outcome following surgery. The differentiation between a RHD and non-RHD aetiology for valve disease nonetheless relied on the opinion of the individual surgical centre and was not confirmed by independent sources nor benchmarked against agreed echocardiographic [5] or pathologic criteria. It is therefore possible that the stratification of RHD and non-RHD aetiology may not have always been accurate. Nevertheless the majority of patients came from a relatively small number of high volume centres which have considerable experience in managing patients with RHD and thus, it would be assumed, significant skill in differentiating RHD and non-RHD related valve disease.

The relatively small number of Indigenous Australian patients in this study is also a limitation when undertaking comparisons with non-Indigenous Australians. This reflects the relatively small size of the Indigenous Australian population, the residual burden of RHD in older non-Indigenous Australians and the fact the database began with only a few centres and has only gradually increased over time [33]. During the early years the sample was likely to have not been representative of surgical experience in RHD in Indigenous patients and therefore surveillance of longer term survival in this group of patients will be required.

The ANZSCTS Database does not collect information regarding pulmonary pressures and particularly the presence of pulmonary hypertension. Pulmonary hypertension has been associated with poorer early post-operative mortality in patients having surgery for mitral regurgitation both in those with and without left ventricular functional impairment [65]. In addition, even in patients with mitral valve disease and no overt pulmonary hypertension detected on echocardiography, it has been shown that in many pulmonary hypertension can be revealed by exercise and this in turn is associated with poorer outcome [66, 67]. Thus our inability to include resting and exercise-related pulmonary hypertension in our analyses may in part explain the lack of importance of NYHA functional class and reduced LVEF, as a predictors of long-term survival.

## Conclusion

We have presented short and long term outcome data relating to 17 227 surgical procedures required for the management of patients with advanced RHD and non-RHD related valve disease. RHD valve surgery patients, compared with non-RHD patients, had a longer period of invasive ventilation, were more likely to be readmitted to hospital, develop an anticoagulant complication and less likely to have a stroke. Independent predictors of short term mortality following RHD-related valve surgery were co-existent chronic kidney disease, length of stay in ICU following surgery, acute kidney injury, anticoagulant complication and requiring re-operation for valve dysfunction. Longer term survival in RHD patients, out to 10 years, was at the upper end of that reported in earlier studies and was poorer in those with co-existent chronic kidney disease and diabetes, and those who required a longer period of ventilation and stay in hospital following surgery. Of note, being an Aboriginal Australian and/or Torres Strait Islander, co-existent chronic disease, pre-existing AF, a greater functional impairment as assessed by NYHA functional class and poorer pre-operative LVEF were not independently associated with outcome.

Thus this large cohort of valve surgery patients demonstrates that short and long term outcomes in Australia are comparable to other countries. Whilst the choice of procedure undertaken for the management of advanced RHD is likely to be best informed by patient preference, the ability to maintain safe anticoagulation and the underlying nature of the valve lesion, we have demonstrated poorer long term survival in those having bioprosthetic valve replacements. This may possibly relate to other factors which we have not assessed or controlled for. Ongoing surveillance of valve surgery in this setting should consider incorporating long-term assessment of the adequacy of anticoagulation, measures of baseline exercise tolerance and detailed measurement of resting and exercise-related pulmonary hypertension. These may provide additional insight into why AF is not an independent predictor of outcome, how neither poorer NYHA class nor LVEF influences survival and why bioprosthetic valves may be associated with poorer long term survival. Together they may better inform how best to manage AF and the timing and nature of surgery for advanced RHD.

## Abbreviations

RHD: Rheumatic heart disease
OR: Odds ratio
CI: Confidence interval
AF: Atrial fibrillation
NYHA: New York Heart Association functional class
LVEF: Left ventricular ejection fraction
ANZSCTS: Australia and New Zealand Society of Cardiac and Thoracic Surgeons
RA: Remoteness area
eGFR: Estimated glomerular filtration rate
MDRD: Modification of diet in renal disease
CABG: Coronary artery bypass grafting
PBV: Percutaneous balloon valvuloplasty
TIA: Transient ischaemic attack
NDI: National death index
SD: Standard deviation
IQR: Interquartile range
HR: Hazard ratio
ARF: Acute rheumatic fever
